# Microshear Bond Strength of Bioactive Materials to Dentin and Resin Composite

**DOI:** 10.1055/s-0042-1756692

**Published:** 2022-10-28

**Authors:** Basma Ahmed, Hamdi H. Hamama, Salah Hasab Mahmoud

**Affiliations:** 1 Operative Department, Faculty of Oral and Dental Medicine, Delta University for Science and Technology, Gamasa, Egypt; 2 Operative Dentistry, Faculty of Dentistry, Mansoura University, Mansoura, Egypt; 3 Restorative Dentistry Dept, New-Mansoura University, New Mansoura, Egypt; 4 Conservative Dentistry Dept, Horus University, New Damietta, Egypt

**Keywords:** bioactive materials, resin-modified glass ionomer, microshear, glass ionomer

## Abstract

**Objectives** The aim of this study was to comparatively evaluate microshear bond strength (μSBS) of bioactive ionic resin composite and resin-modified glass ionomer liner (RMGI) to dentin and resin composite.

**Materials and Methods** The enamel of 11 posterior molar teeth was removed to expose dentin and then placed in acrylic blocks. Each specimen received three microcylindrical Tygon tubes filled with bioactive ionic resin composite (Activa Bioactive base/liner ( **Pulpdent** , MA, USA)), RMGI (Riva light cure SDI LTD, Bayswater, Australia), and resin composite (Filtek Z350xt, MN, USA). Composite discs ( *n* = 11) were fabricated from nanofilled resin composite (Filtek Z350xt) and then fixed in acrylic blocks. Each specimen received two microcylindrical Tygon tubes filled with Activa Bioactive base/liner and Riva RMGI. All specimens were mounted individually to universal testing machine for μSBS test. Failure modes were analyzed using stereomicroscope and scanning electron microscope.

**Results** Filtek Z350xt nanofilled resin composite showed the highest μSBS values. No statistical significant difference was found between Activa Bioactive and Riva RMGI ( *p* > 0.05).

**Conclusion** Bioactive ionic resin composite liner exhibited similar bond strength as RMGI to dentin and resin composite.

## Introduction

Maintaining pulp vitality during dental caries treatment is important to preserve tooth integrity and health of its supporting tissues. 1 Complete caries excavation of deep carious lesions is highly questionable due to the risk of pulp exposure and it may cause weakening of tooth structure, thus compromising the success of dental treatment. 2 In order to avoid pulp exposure and to preserve as much as possible of tooth structure, the modern concept of “minimal intervention dentistry” calls for conservative elimination of carious lesion. 3

Selective caries excavation involves removal of the outer contaminated infected dentin layer, while maintaining the deeper layer of affected carious dentin, which can be remineralized. This concept is based on substantial evidence that removal of all deep carious lesions is not required for a successful dental management, provided that the restoration can be sealed effectively from oral environment. 2 As when cariogenic bacteria become isolated from their nutritional source by a restoration that has sufficient integrity, they either die or remain quiescent and thus pulp could stay vital. 4

Many bacteriostatic, bactericidal, and remineralizing materials have been applied to the remaining partially demineralized dentin after selective caries excavation aiming to its remineralization and forming hard bacterial free dentin; however, there is no consensus found on which material would be the most effective. 5 Calcium hydroxide has been extensively considered the gold standard material for dentin remineralization. However, this material has some noticeable drawbacks including degradation by time, insufficient adherence to dentinal walls, low mechanical properties, and high solubility. Another concern about calcium hydroxide would be tunnel defect formation in reparative dentin under the lining material. Therefore, calcium hydroxide is no longer seems to be the best possible material of choice. 6

Nowadays, calcium hydroxide has been replaced by other lining materials that result in more predictable clinical outcomes such as glass ionomer cements and resin-modified glass ionomer (RMGI) liners. 6 RMGI liners offer the merits of chemical adhesion to tooth structure, fluoride release, and antibacterial activity. 7 In addition, using RMGI as dentin substitute material may provide a sort of “stress absorption” effect at the bonding interface. This has been advocated to avoid the development of stresses at the dentin bonded interface and to reduce gap formation, microleakage, and degradation by time. 8

Recently, bioactive materials have been continuously emerging in the dental market adding beneficial properties to that available in present dental materials. A new bioactive lining material, known as Activa Bioactive base/liner, has been recently introduced to dental field. Activa Bioactive is considered the first dental resins with a bioactive ionic resin matrix that releases and recharges an abundant amount of calcium, phosphate, and fluoride ions and reacts to the continuous pH changes in the mouth. This material consists of ionic resin matrix, a shock absorbing resin component, and bioactive fillers that mimic the physical and chemical properties of natural teeth. It has the ability to make a chemical bond with tooth structure. Therefore, it provides a good seal against microleakage. According to the manufacturer, this material is self-adhesive and does not need additional pretreatment before its application on dentin. 9

Pulp lining materials have a close proximity with the pulp tissue and thus should be nontoxic and biocompatible. 10 A previous study performed by Abou ElReash et al 11 stated that Activa Bioactive had a high degree of biocompatibility and it decreased the intensity of inflammation. This was also confirmed by Bakir et al 12 who reported that Activa Bioactive base/liner is a biocompatible material, as it showed successful tissue response. It was reported that Activa Bioactive material had the potential to stimulate biomineralization at the same level as MTA, Biodentine, and TheraCal LC on the basis of releasing the same amount of Ca and OH ions. 13

Activa Bioactive being a newly introduced material has limited data available on it. Therefore, this study aimed to assess and compare bond strength of Activa Bioactive with RMGI liner to dentin and resin composite restorative material. The null hypothesis tested was that there would be no significant difference in microshear bond strength (μSBS) of both lining materials to dentin and resin composite restorative material.

## Materials and Methods

The full description of materials used in the current study is illustrated in Table 1 .

**Table 1:** Materials used in the study

Materials	Type	Manufacture	Composition	Batch no.
Filtek Z350 xt	Nanofilled resin composite	3M ESPE MN, USA	Matrix: Bis-GMA, UDMA, TEGDMA, PEGDMA, Bis-EMA. Filler: Combination of nonagglomerated/nonaggregated 20 nm silica filler, nonagglomerated/nonaggregated 4 to 11 nm zirconia filler, and aggregated zirconia/silica cluster filler	NC93014
Single bond Universal	Universal adhesive	3M ESPE	MDP phosphate monomer, dimethacrylate resin, HEMA, filler, ethanol, water, initiators, silane, Vitrebond copolymer.	517571
Activa Bioactive Base/Liner	Bioactive ionic resin with reactive glass filler	Pulpdent ( **Pulpdent** , MA, USA)	Blend of diurethane and other methacrylates with modified polyacrylic acid, silica, amorphous, sodium fluoride	181213
Riva Light Cure	Resin-modified glass ionomer	SDI Limited Bayswater, Australia	2-hydroxyethyl methacrylate, acrylic acid homopolymer, dimethacrylate cross-linker, acidic monomer, tartaric acid, glass powder	J2102033
Riva Conditioner	Polyacrylic acid conditioner	SDI Limited	Polyacrylic acid 25–30% by wt	200250

Abbreviations: Bis-EMA, bisphenol A ethoxylate dimethacrylate; Bis-GMA bisphenol A-glycidyl dimethacrylate; HEMA, 2-hydroxyethyl methacrylate; MDP, methacryloyloxydecyl dihydrogen phosphate; PEGDMA, Polyethylene glycol dimethacrylate; TEGDMA, triethylene glycol dimethacrylate; UDMA, urethane dimethacrylate.

## Methods

### Teeth Selection

Eleven extracted human permanent molars were selected from healthy individuals after obtaining their consent. All teeth were examined macroscopically and microscopically (20× magnification) to exclude dental caries, cracking, and fracture. A hand scaler was used to remove any soft tissue remnant or hard deposits. The selected teeth were then washed under running water and placed in 0.5% solution of chloramine-T for 2 days for disinfection. 14 Finally, teeth were polished by using pumice rubber cups, then stored in distilled water for 24 hours at 37°C in an incubator (BTC, Model: BT1020, Cairo, Egypt).

### Specimen Preparation

The enamel of the selected molars was removed by sectioning the teeth at the occlusal third of the crown with a slow speed diamond saw (ISOMET 4000; Buehler, Lake Bluff, Illinois, United States) under water cooling system in order to prepare a flat superficial surface of dentin. Each dentin specimen was mounted vertically in polyvinyl chloride rings (PVC, 1.4 × 2.5 cm) filled with auto-polymerizing acrylic resin (Acrostone, Egypt) where the occlusal surface of teeth facing upward. After acrylic resin setting, the specimens were removed from the PVC molds. The occlusal surfaces of molars were polished by using 600-grit silicon carbide paper for 60 seconds in order to create standardized smear layer. 15

**Fig. 1:**
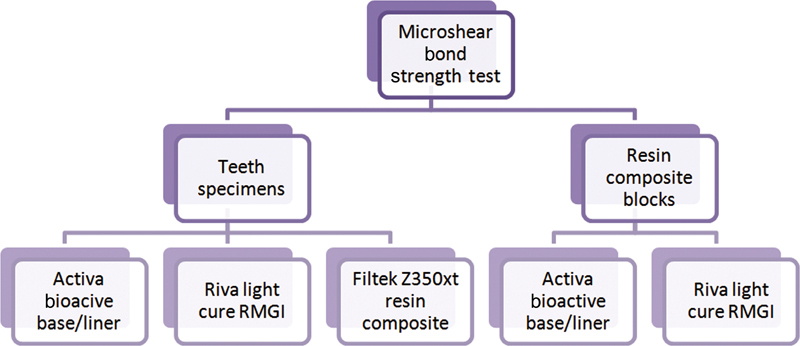
Flowchart representing the experimental design. RMGI, resin-modified glass ionomer liner.

Each tooth received three microcylindrical plastic tubes (Tygon tubes) that had 1 mm internal diameter and 2 mm height. The Tygon tubes were filled with different restorative materials as follows: Filtek Z350xt resin composite, Riva light cure RMGI, and Activa Bioactive base/liner ( Fig. 1 ).

Regarding resin composite, the universal bonding agent (Single Bond Universal) was first applied on dentin surface by microbrush and rubbed for 20 seconds. The bonding agent was gently air dried for 5 seconds followed by light curing for 10 seconds. The Tygon tubes were held by tweezer, fixed on dentin surface and filled with composite resin (Filtek Z350xt), then light cured for 20 seconds following manufacturer's instructions.

For Riva RMGI, dentin was first conditioned utilizing Riva Conditioner (poly acrylic acid conditioner) for 10 seconds, then conditioner was rinsed thoroughly with water. The excess water was air dried keeping dentin moist. Riva RMGI was then applied in Tygon tubes that were fixed on dentin surface and the material was light cured for 20 seconds. For Activa Bioactive, the material was applied directly in Tygon tubes fixed on dentin surface, agitated into the dentin for 20 seconds by using thin dental instrument, and then light cured for 20 seconds.

The Tygon tubes were removed from around the restorative materials leaving 11 dentin specimens with 33 microcylindrical tubes of set material. Two parallel cuts were made longitudinally in Tygon tubes to facilitate their removal from around set material. The microcylindrical tubes of material were checked with stereomicroscope for detection of interfacial defects. Finally, the specimens were stored in distilled water 24 hours before μSBS testing.

In addition, 11 composite discs were prepared from nanofilled resin composite (Filtek Z350xt) using a split plastic mold of 2 mm height and 10 mm diameter. The resin composite was placed into the mold by using gold-plated instrument then pressed against a Mylar strip and a glass slide for the material protection and to ensure a smooth surface. The resin composite was light cured for 20 seconds according to manufacturer's instruction. After removal of the mold, only one side of composite discs was finished using finishing discs. The composite blocks were then placed in PVC rings filled with auto-polymerizing acrylic resin that were removed after acrylic setting. Each block received two Tygon tubes filled with Riva light cure RMGI and Activa Bioactive base/liner ( Fig. 1 ).

Each material was inserted in Tygon tubes that were fixed on composite surface and the materials were light cured for 20 seconds. The Tygon tubes were then removed from around the restorative materials leaving 11 composite blocks with 22 microcylindrical tubes of set material.

### Microshear Bond Strength Testing

The mechanical μSBS test was performed in a universal testing machine (Instron 3345, Canton, Massachusetts, United States). Each specimen with the microcylindrical tubes of tested materials was placed in the lower fixed compartment of the universal testing machine. A thin orthodontic wire (diameter 0.14 mm) was looped around each microcylindrical tube as close as possible to its base. The wire was aligned with the loading axis of the upper movable compartment of the testing machine to ensure proper distribution of shear load. 14 Shear force was applied to each specimen at a crosshead speed of 0.5mm/min until failure occurred. The μSBS values (expressed in MPa) were calculated from the maximum failure load (expressed in Newton) divided by the bonded surface area (mm ^2^ ).

### Failure Mode Analysis

The failure mode was identified by examining all the debonded surface specimens under a stereomicroscope (SZ-PT, Olympus, Japan) at approximately 40x magnification. The failures were classified as following: adhesive (failure at interface), cohesive, and mixed (combination of adhesive and cohesive failure). Representative samples from each fracture type were sputter-coated with gold and examined by scanning electron microscope (JSM-6510LV SEM, JEOL Ltd, Tokyo, Japan) at approximately 35x magnification for the verification of the fracture pattern.

## Results

Statistical Software Package Program (SPSS, V.22, IBM Armonk, New York, United States) was used for the statistical analysis of the collected data. The data were tabulated and statistically evaluated using one-way analysis of variance followed by Tukey honestly significant difference post-hoc multiple comparison tests. The level of significance was set at *p* <0.05.

According to the results obtained from dentin specimens ( Table 2 ), resin composite showed the greatest μSBS value (20.24 ± 3.46 MPa), while Activa Bioactive showed the lowest value (16.23 ± 2.63 MPa). There was no statistical significant difference detected between Activa Bioactive and Riva RMGI ( *p* > 0.05). However, statistical significant difference was found between the resin composite and both liners ( *p* < 0.05). Regarding resin composite blocks, the results revealed that no significant difference was found between Activa Bioactive and Riva RMGI ( Table 3 ).

**Table 2:** Mean (± SD) microshear bond strength of tested materials of dentin specimens

Materials	Mean (MPa) ± SD
Resin composite	20.24 ± 3.46 ^a^
Activa Bioactive	16.23 ± 2.63 ^b^
Riva RMGI	17.26 ± 2.16 ^b^

Abbreviation: SD, standard deviation.

*Different letters indicate significant difference at level of significance *p* < 0.05.

**Table 3:** Mean (± SD) microshear bond strength of tested materials of composite blocks

Materials	Mean (MPa) ± SD
Activa Bioactive	11.75 ± 2.03
Riva RMGI	13.00 ± 1.82

Abbreviation: SD, standard deviation.

The allocation of failure modes of fractured dentin and composite specimens is illustrated in Tables 4 and 5 . Descriptive stereomicroscope and SEM images showing the different failure mode patterns are displayed in Table 6 . For dentin specimens, the number of adhesive failures was low in resin composite which revealed the highest μSBS mean values, while the adhesive failure number was high in Activa Bioactive which had the lowest μSBS mean values. Regarding composite blocks, Activa Bioactive revealed higher number of adhesive failures than Riva RMGI. There was no cohesive failure mode recorded among all composite specimens.

**Table 4:** Failure modes of dentin specimen groups

Failure mode	Groups		
	Resin composite	Active Bioactive	Riva RMGI
Adhesive	9% (1)	45% (5)	27% (3)
Cohesive	36% (4)	0	9% (1)
Mixed	55% (6)	55% (6)	64% (7)

Abbreviation: RMGI, resin-modified glass ionomer liner.

**Table 5:** Failure modes of composite discs specimen groups

Failure mode	Groups	
	Active Bioactive	Riva RMGI
Adhesive	45% (5)	36% (4)
Cohesive	0	0
Mixed	55% (6)	64% (7)

Abbreviation: RMGI, resin-modified glass ionomer liner.

**Table 6:** Descriptive stereomicroscope and SEM images showing different failure mode patterns

Mode of failure	Stereomicroscope images	SEM images
Adhesive		
Cohesive		
Mixed		

Abbreviation: SEM, scanning electron microscope.

## Discussion

An effective adhesion of dental material to tooth structure is essential to prevent the formation of secondary caries, microleakage, marginal discoloration, and subsequent pulpal damage. A durable bond of dental biomaterials to tooth structure is important to accomplish good mechanical as well as biological and esthetic properties. μSBS test has an essential clinical importance, because the majority of dislodging forces have a shearing effect at the tooth restoration interface. 16

The result of this study revealed that Filtek Z350xt resin composite had significantly higher μSBS compared to the other tested materials (Activa Bioactive and Riva RMGI). This could be attributed to the use of single bond universal adhesive which contains 10-methacryloyloxydecyl dihydrogen phosphate (10-MDP) that bond chemically to dentin. 17 This was supported by Yoshida et al. 18 who reported that an effective chemical interaction occurs between MDP and hydroxyapatite forming a stable nano-layer that could form a stronger phase at the adhesive interface, thus increases the mechanical strength of the adhesive interface. Moreover, the stable MDP-calcium salt deposition along with nano-layering could explain the high bond stability which has been previously proven both in laboratory and clinical researchs. 19 20

This result was also in agreement with Latta et al 21 who reported that the resin composite had the highest μSBS value when compared to Activa Bioactive and RMGI. It was suggested that micromechanical retention had greater effect on dentin than did chemical bonding on the same substrate. As micromechanical retention is more essential for the resistance of mechanical stresses, while chemical bonding enhances the resistance to hydrolytic degradation. 22 A previous laboratory study conducted by Tohidkhah et al 23 also reported that resin composite had higher shear bond strength than Activa Bioactive base/liner and RMGI.

In contrast, Rifai et al 24 disagreed with the present study where Activa Bioactive had similar bond strength as Filtek Z350xt resin composite. Their explanation was based on ionic resin component in Activa Bioactive that contains phosphate acid groups with antimicrobial properties which enhance the interaction between the resin and the reactive glass fillers and improve the interaction with tooth structure. As an ionic interaction binds the resin to the tooth minerals, creating a strong complex of resin-hydroxyapatite.

Activa Bioactive showed similar μSBS as RMGI, where there was no statistical significant difference between the both liners. This can be ascribed to the similarity in composition and properties between the two lining materials. Some studies 25 26 considered Activa Bioactive base/liner as an altered RMGI material. Due to the reduced studies comparing μSBS of Activa and RMGI, there was no studies agreed with the present study result. Conversely, Latta et al 21 and Tohidkhah et al 23 stated that RMGI had higher bond strength than Activa Bioactive. They attributed their results to the low self-adhesive potential of Activa Bioactive to dentin when compared to other self-adhesive materials.

There is a correlation between bond strength and mode of failure. 27 According to Gupta and Mahajan, 28 the higher the bond strength, the lower the number of adhesive failure and the higher the number of mixed and cohesive failure. The results of failure mode analysis revealed that the higher μSBS value (resin composite) was associated with mixed and cohesive failures, but the lower bond strength value (Activa Bioactive) was mostly associated with adhesive failures. This result is consistent with Sabatini 29 who reported that mixed failure was corresponding to the highest bond strength value, while adhesive failure was corresponding to the lowest bond strength value. On the other hand, previous studies 30 31 stated that no direct correlation was found between bond strength and failure mode, where mixed and cohesive failure modes were not necessarily associated with high bond strength values.

Finally, this experimental study evaluated and compared μSBS of Activa Bioactive base/liner with RMGI to dentin and resin composite restorative material. Since the results showed Activa Bioactive and RMGI had similar microshear bond strength to dentin and resin composite, therefore, the null hypothesis was accepted.

## Conclusion

Bioactive ionic resin composite liner exhibited similar bond strength as RMGI to dentin and resin composite.
